# The dynamic systemic immuno-inflammatory index: a novel predictor of neoadjuvant chemotherapy response and outcome in high-risk pediatric neuroblastoma

**DOI:** 10.3389/fonc.2025.1671233

**Published:** 2025-11-05

**Authors:** Chenglong Zhang, Xiaoyu Wang, Huizhong Niu, Pengju Zhang, Jianlei Geng

**Affiliations:** ^1^ General Surgery Department, Hebei Children’s Health and Disease Clinical Medical Research Center, Hebei Children’s Hospital, Shijiazhuang, Hebei, China; ^2^ Anestesiology Department, Hebei Provincial People’s Hospital, Shijiazhuang, Hebei, China

**Keywords:** high-risk neuroblastoma, neoadjuvant chemotherapy, systemic immune-inflammation index, dynamic changes, overall survival

## Abstract

**Background:**

High-risk neuroblastoma (HR-NB) has a high mortality rate and a long-term survival rate below 50%. Neoadjuvant chemotherapy (NAC) is essential for HR-NB treatment. However, real-time treatment response biomarkers are lacking. The Systemic Immune-Inflammation Index (SII) shows promise as a prognostic tool for various malignancies. This study explored the relationship between dynamic SII changes during NAC and treatment responses and prognosis in HR-NB.

**Methods:**

This retrospective study analyzed 50 HR-NB patients’ clinical data from January 2013 to November 2024. Peripheral blood samples were collected before and after each cycle of neoadjuvant chemotherapy (NAC) to calculate SII values.

**Results:**

Significant differences in SII changes were found among treatment response groups (P < 0.05). Spearman analysis showed a strong positive correlation between ΔSII and tumor diameter change rate (r = 0.606, P < 0.001). Regression analyses indicated ΔSII was an independent predictor of tumor diameter change (β = 0.07, 95% CI: 0.05-0.10, P < 0.001) and chemosensitivity (OR = 0.00, 95% CI: 0.00-0.03, P = 0.010). ΔSII was also an independent prognostic factor for EFS and OS (HR = 1.35 and 1.41, P < 0.05) with high predictive accuracy (AUC: 0.766-0.932).

**Conclusion:**

Dynamic SII changes during NAC are significantly linked to treatment response and prognosis in HR-NB, offering a new perspective for precision treatment and prognosis evaluation.

## Introduction

Neuroblastoma (NB), a neurocrest-derived tumor, accounts for 8–10% of all pediatric cancers (1). High-risk neuroblastoma (HR-NB) is a leading cause of childhood cancer mortality, with a long-term survival rate of less than 50% (1, 2). The current International Neuroblastoma Risk Group (INRG) stratification system relies on static indicators such as age, stage, and MYCN amplification, yet it fails to predict treatment responses in real time (3). Neoadjuvant chemotherapy (NAC), a key part of standard therapy, has its early efficacy directly impact surgical resection rates and survival outcomes (4). Yoo et al. demonstrated that patients with a >60% reduction in tumor volume after 2–3 cycles of NAC had a 5-year recurrence-free survival rate of 83%, significantly higher than that of poor responders (51%) (5). However, imaging assessments have certain lagging effects (6). There is an urgent need to obtain quantifiable biological response biomarkers in the early stages of chemotherapy to guide individualized treatment adjustments.

Systemic inflammatory responses play a crucial role in tumor progression by promoting angiogenesis, metastasis, and immune evasion via modulation of the tumor microenvironment (7). Recently, systemic inflammatory markers based on peripheral blood cell counts have become important prognostic assessment tools due to their noninvasive and easily obtainable nature. The Systemic Immune-Inflammation Index (SII), which integrates neutrophils, platelets, and lymphocytes, more comprehensively reflects the host’s inflammatory status (8). SII has shown significant prognostic value in various adult cancers and has also been validated in pediatric cancers (9–13). Precision therapy for HR-NB requires consideration of both tumor biological characteristics and treatment responses (14). However, there is currently a lack of dynamic parameters reflecting changes in the host’s inflammatory status during treatment. Moreover, no studies have explored the correlation between dynamic SII changes and NAC responses.

This study aims to analyze the dynamic changes in SII during NAC in children with HR-NB and to investigate the relationship between changes in peripheral blood SII before and after NAC and treatment responses as well as long-term prognosis.

## Methods

### Patient selection

This retrospective study analyzed the clinical data of consecutive patients diagnosed with high-risk neuroblastoma and admitted for surgery at our hospital between January 2013 and November 2024. Inclusion criteria were pathologically confirmed neuroblastoma, defined as high-risk according to the International Neuroblastoma Risk Group (INRG) classification system, undergone radical tumor resection, and had complete preoperative blood component test results and follow-up data. Exclusion criteria were severe infection or immunodeficiency, other malignancies, incomplete serological or follow-up data. Finally, 50 patients were included. All patients received uniform induction chemotherapy according to the Children’s Oncology Group (COG) ANBL0532 regimen. It is important to note that due to resource constraints at our center during the study period, none of the patients underwent subsequent autologous stem cell transplantation (ASCT) or received anti-GD2 immunotherapy after chemotherapy and surgery.

### Data collection

We retrospectively collected patients’ demographic, pathological, and laboratory data. As part of routine clinical practice, fasting venous blood samples were collected within 1 week prior to neoadjuvant chemotherapy (NAC), and after the first, second, third, and fourth cycles of NAC. These time points were defined as T0, T1, T2, T3, and T4, with T0 as the baseline. All patients showed no fever (axillary temperature <37.28°C) or active infection/chronic inflammation at blood collection. Neutrophil, lymphocyte, and platelet counts were extracted from the test results. The Systemic Immune-Inflammation Index (SII) was calculated as: SII = platelet count × neutrophil count/lymphocyte count. The change rate of SII from before NAC (T0) to after NAC (T4) was defined as ΔSII%. Treatment response after NAC was defined as: complete response (CR), partial response (PR), progressive disease (PD), or stable disease (SD). All participants signed informed consent, and the study was approved by the Medical Research Ethics Committee of Hebei Children’s Hospital (approval No.: 202430).

### Follow-up

All patients were followed up every 3 months for the first 3 years after surgery, then every 6 months. During follow-up, clinical history was collected, physical examinations were performed, peripheral tumor biomarker levels were measured, and abdominal/pelvic CT scans or ultrasounds were conducted, based on the NCCN Oncology Clinical Practice Guidelines. Given that all patients had primary tumors originating from the adrenal gland (an abdominal site), surveillance for local recurrence was performed with abdominal/pelvic CT or ultrasound. For the evaluation of distant metastatic disease, MIBG scintigraphy was performed at regular intervals and whenever there was clinical suspicion of recurrence. Overall survival (OS) was calculated from initial treatment to death. Event-free survival (EFS) was calculated from initial treatment to first disease progression/recurrence.

### Statistical analysis

Statistical analysis was performed using GraphPad Prism 9.5.1.733 for Windows and R (version 4.2.2). Continuous variables were expressed as mean ± standard deviation or median (interquartile range [IQR]), and categorical variables as frequency and percentage, with comparisons made using the chi-square test. Spearman correlation analysis was used to assess the association between ΔSII% and primary tumor diameter reduction rate. Univariate and multivariate linear regression analyses examined the impact of ΔSII% on primary tumor reduction rate. Univariate and multivariate logistic regression analyses assessed the effect of ΔSII% on treatment response. Kaplan-Meier survival curves analyzed survival differences across ΔSII% groups. Univariate and multivariate Cox regression analyses constructed prognostic models. Time-dependent ROC curves evaluated the predictive ability of ΔSII for different survival periods.

## Results

### Baseline characteristics and outcomes

Among the 50 participants, 29 (58.0%) were in the survival group and 21 (42.0%) in the mortality group (**Table 1**). The mortality group had significantly lower median overall survival (OS) (20.0 [IQR, 16.0–37.0] months vs. 47.0 [38.0–63.0] months; P = 0.002) and event-free survival (EFS) (16.0 [11.0–32.0] months vs. 47.0 [36.0–61.0] months; P = 0.003) than the survival group. No significant between-group differences were found for year (P = 0.453) or tumor size (P = 0.118). The mortality group had higher baseline systemic immune-inflammation index (SII) values (629.8 [549.3–1115.5] vs. 441.0 [219.0–733.9]; P = 0.031) and post-treatment SII (946.6 [716.1–1222.8] vs. 170.8 [68.2–270.8]; P<0.001). The change in SII (ΔSII) differed significantly between groups (0.31 [0.18–0.90] vs. −0.57 [−0.75 to −0.25]; P<0.001), as did the change in tumor size (ΔSize) (0.04 [−0.06–0.12] vs. −0.25 [−0.37 to −0.07]; P<0.001). No significant differences were noted in year* (P = 0.851), sex (P = 0.704), pathologic classification (P = 0.373), or chromosomal deletion (P = 0.591). However, the mortality group had a higher proportion of MYCN amplification (52.4% vs. 10.3%; P = 0.001) and a lower proportion of patients with therapeutic response (4.8% vs. 48.3%; P<0.001).

**Table 1:** Basic characteristics and differential analysis.

Variables	Total (n = 50)	Survival Group (n = 29)	Mortality Group (n = 21)	Statistic	P
Year, M (Q_1_, Q_3_)	36.00 (12.75, 60.00)	36.00 (24.00, 60.00)	40.00 (12.00, 52.00)	Z=-0.75	0.453
Year*(month), n(%)				χ²=0.04	0.851
< 18	15 (30.00)	9 (31.03)	6 (28.57)		
≥ 18	35 (70.00)	20 (68.97)	15 (71.43)		
Sex, n(%)				χ²=0.14	0.704
Male	27 (54.00)	15 (51.72)	12 (57.14)		
Female	23 (46.00)	14 (48.28)	9 (42.86)		
Tumour size (cm), M (Q_1_, Q_3_)	9.00 (7.17, 11.25)	9.60 (8.00, 12.20)	8.30 (7.00, 10.00)	Z=-1.56	0.118
Pathologic, n(%)				χ²=0.79	0.373
Neuroblastoma	42 (84.00)	26 (89.66)	16 (76.19)		
Ganglioneuroblastoma, nodular	8 (16.00)	3 (10.34)	5 (23.81)		
MYCN, n(%)				χ²=10.68	0.001
No	36 (72.00)	26 (89.66)	10 (47.62)		
Yes	14 (28.00)	3 (10.34)	11 (52.38)		
11q Deletion, n(%)				χ²=0.29	0.591
No	41 (82.00)	25 (86.21)	16 (76.19)		
Yes	9 (18.00)	4 (13.79)	5 (23.81)		
SII(T0), M (Q_1_, Q_3_)	563.27 (299.02, 835.47)	440.99 (219.04, 733.93)	629.78 (549.29, 1115.49)	Z=-2.15	0.031
SII(T4), M (Q_1_, Q_3_)	495.34 (113.58, 937.47)	170.80 (68.19, 270.79)	946.56 (716.09, 1222.75)	Z=-5.03	<.001
ΔSII, M (Q_1_, Q_3_)	-0.19 (-0.61, 0.31)	-0.57 (-0.75, -0.25)	0.31 (0.18, 0.90)	Z=-4.95	<.001
ΔSize, M (Q_1_, Q_3_)	-0.09 (-0.34, 0.04)	-0.25 (-0.37, -0.07)	0.04 (-0.06, 0.12)	Z=-4.20	<.001
Therapeutic Response, n(%)				χ²=10.98	<.001
SD or PD	35 (70.00)	15 (51.72)	20 (95.24)		
CR or PR	15 (30.00)	14 (48.28)	1 (4.76)		

MYCN, MYCN gene amplification; 11q Deletion, Chromosome 11q deletion; SII, Systemic Immune-Inflammation Index; ΔSII, The change rate of the systemic inflammatory index (SII); ΔSize, The change rate of the tumour size; CR, Complete Response; PR, Partial Response; SD, Stable Disease; PD, Progressive Disease.

Year*, Age is presented as a category.

### Dynamic changes in SII

**Figure 1:**
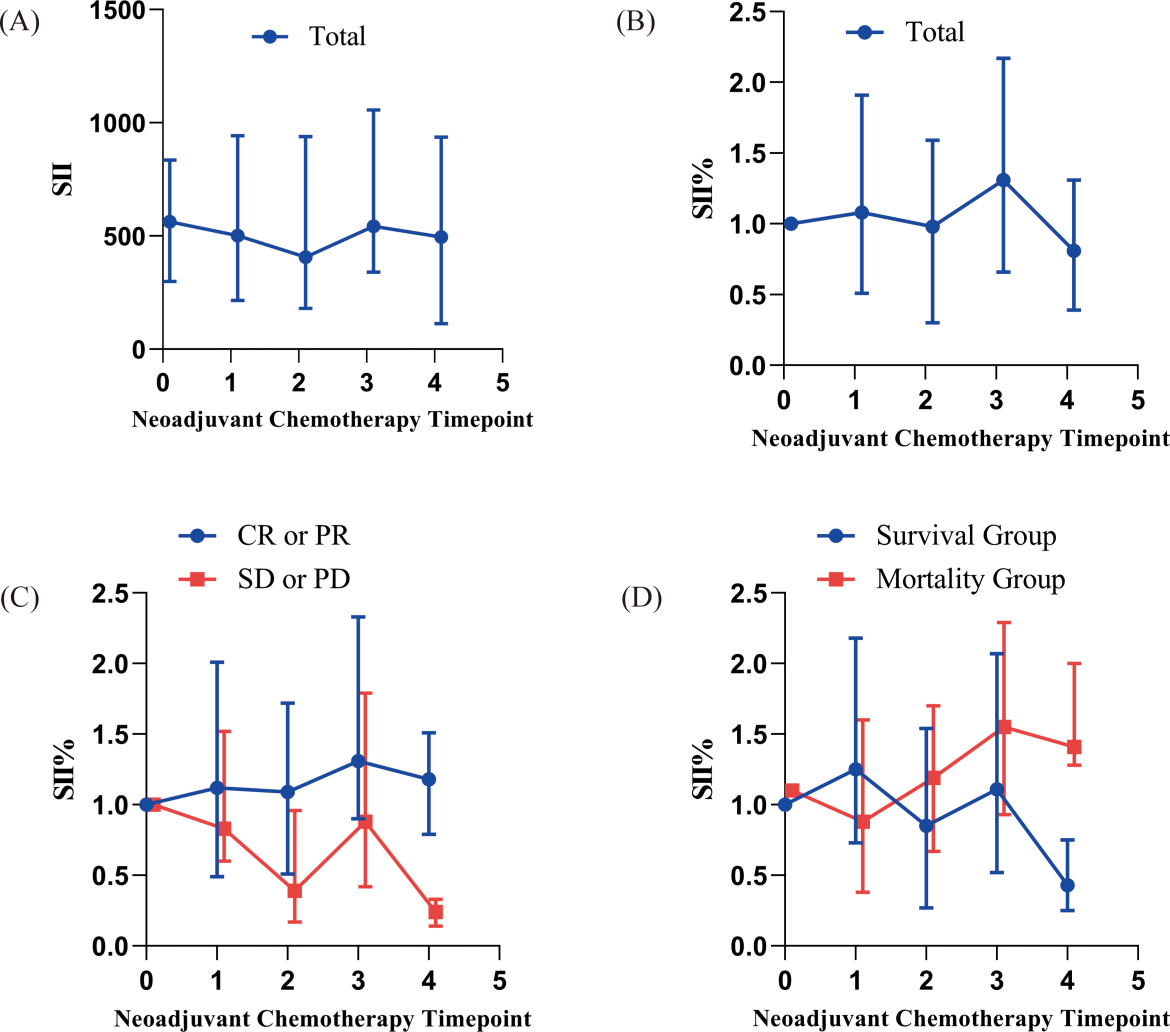
Dynamic changes in the systemic immune-inflammation index (SII) during neoadjuvant chemotherapy. **(A, B)** Line charts showing the absolute and relative values of SII changes across chemotherapy cycles (T0-T4) in all patients. **(C)** Comparison of SII change patterns between patients with different treatment responses (complete/partial response vs. stable/progressive disease). **(D)** Comparison of SII change patterns between patients with different survival statuses (survival vs. mortality).

**Figures 1A, B** show the line graphs of the absolute and relative changes in SII from T0 to T4 for all patients. Results indicated a marked downward trend in SII after neoadjuvant chemotherapy (NAC) compared to before NAC. **Figure 1C**, grouped by treatment response (CR/PR vs. SD/PD), revealed significant differences in SII change patterns between the two groups (P < 0.05). The CR/PR group showed a gradual decrease in SII during chemotherapy, while the SD/PD group exhibited no significant change or an upward trend. **Figure 1D**, grouped by survival status (survival vs. mortality), indicated that the survival group had a greater overall decrease in SII during chemotherapy, whereas the mortality group showed minimal change or an upward trend (P < 0.05).

### Correlation between SII changes and treatment response

**Figure 2:**
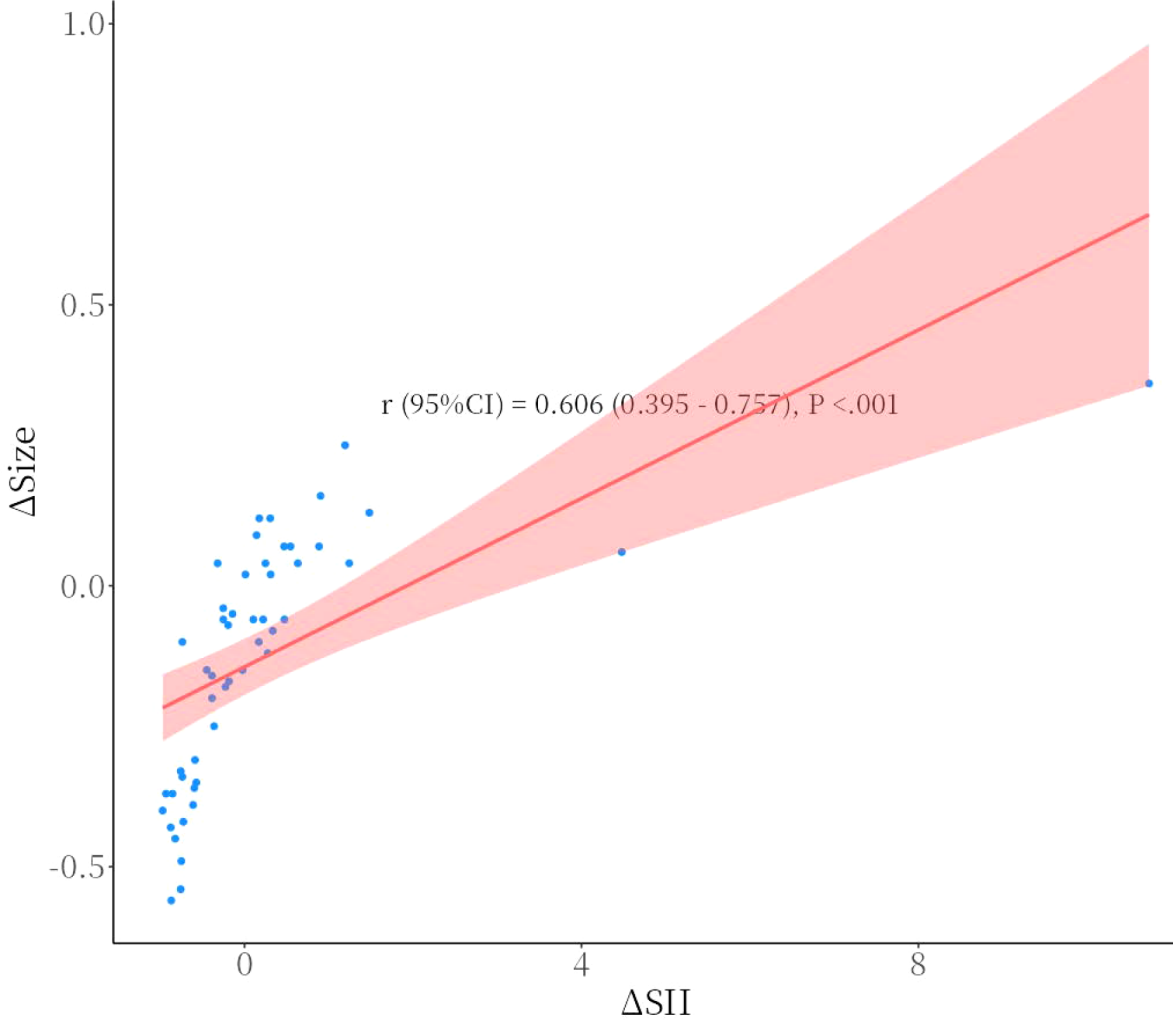
Spearman correlation analysis between the change rate of the systemic immune-inflammation index (ΔSII) and the reduction rate of the primary tumor diameter.

To evaluate the correlation between dynamic SII changes and chemosensitivity in children with high-risk neuroblastoma, correlation and regression analyses were performed. Univariate analysis using Spearman correlation analysis showed a significant positive correlation between SII change rate (∆SII) and primary tumor diameter change rate (r=0.606, 95%CI: 0.395-0.757, P<0.001)(**Figure 2**), indicating that a reduction in SII during chemotherapy was closely related to a reduction in primary tumor diameter, implying a potential association with chemosensitivity. Further multivariate analysis using linear regression analysis (**Table 2**) indicated that when the primary tumor diameter change rate was the dependent variable, ∆SII was an independent influencing factor (β=0.07, 95% CI: 0.05-0.10, P < 0.001). Similarly, in logistic regression analysis (**Table 3**), with treatment response (dichotomous) as the dependent variable and incorporating factors such as ∆SII, age, sex, MYCN gene amplification, and chromosomal deletion, the OR value for ∆SII was 0.00 (95% CI: 0.00-0.03, P = 0.010), suggesting that ∆SII is an independent predictive factor for chemosensitivity. These results indicate that the ∆SII before and after neoadjuvant chemotherapy can effectively predict the chemosensitivity of children with high-risk neuroblastoma.

**Table 2:** Linear regression analysis of SII change rate(ΔSII)and tumor size change rate (ΔSize).

Variables	Univariate analysis		Multivariate analysis	
	β (95%CI)	P	β (95%CI)	P
Year(month)*				
< 18	0.00 (Reference)			
≥ 18	0.01 (-0.12 ~ 0.14)	0.901		
Sex				
Male	0.00 (Reference)			
Female	-0.04 (-0.16 ~ 0.08)	0.493		
Pathologic				
Neuroblastoma	0.00 (Reference)			
Ganglioneuroblastoma, nodular	-0.00 (-0.17 ~ 0.16)	0.966		
MYCN				
No	0.00 (Reference)			
Yes	0.12 (-0.01 ~ 0.25)	0.080		
11q Deletion				
No	0.00 (Reference)			
Yes	0.07 (-0.08 ~ 0.23)	0.363		
Tumour Size	-0.00 (-0.02 ~ 0.02)	0.964		
ΔSII	0.07 (0.05 ~ 0.10)	<.001	0.07 (0.05 ~ 0.10)	<.001

CI, Confidence Interval; MYCN, MYCN gene amplification; 11q Deletion, Chromosome 11q deletion; SII, Systemic Immune-Inflammation Index; ΔSII, The change rate of the systemic inflammatory index (SII). Year*, Age is presented as a category.

**Table 3:** Logistic regression analysis of SII change rate (ΔSII) and therapeutic response.

Variables	Univariate analysis		Multivariate analysis	
	β (95%CI)	P	β (95%CI)	P
Year(month)*				
< 18	1.00 (Reference)			
≥ 18	0.80 (0.22 ~ 2.94)	0.737		
Sex				
Male	1.00 (Reference)			
Female	1.52 (0.45 ~ 5.14)	0.497		
Pathologic				
Neuroblastoma	1.00 (Reference)			
Ganglioneuroblastoma, nodular	0.74 (0.13 ~ 4.19)	0.737		
MYCN				
No	1.00 (Reference)			
Yes	0.29 (0.06 ~ 1.53)	0.145		
11q Deletion				
No	1.00 (Reference)			
Yes	0.24 (0.03 ~ 2.13)	0.200		
Tumour Size	1.06 (0.90 ~ 1.26)	0.487		
ΔSII	0.00 (0.00 ~ 0.03)	0.010	0.00 (0.00 ~ 0.03)	0.010

CI, Confidence Interval; MYCN, MYCN gene amplification; 11q Deletion, Chromosome 11q deletion; SII, Systemic Immune-Inflammation Index; ΔSII, The change rate of the systemic inflammatory index (SII). Year*, Age is presented as a category.

### Correlation between dynamic SII changes and survival prognosis

**Figure 3:**
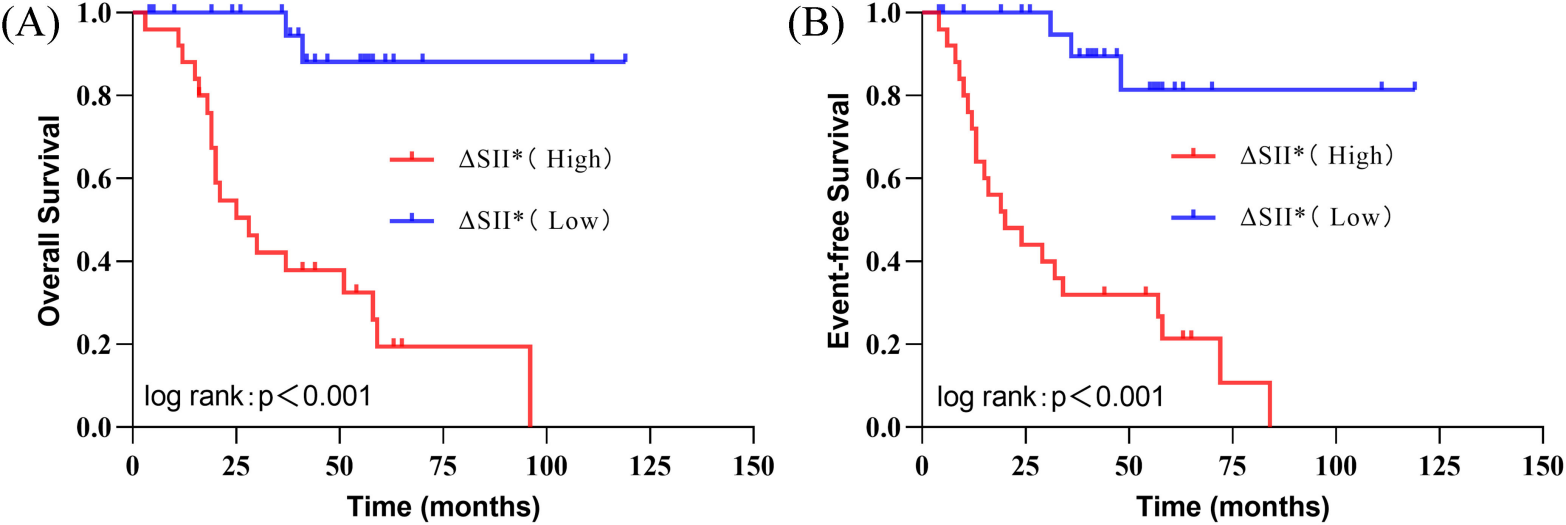
Kaplan-Meier survival curves for event-free survival (EFS) **(A)** and overall survival (OS) **(B)** in patients with low and high ΔSII groups.

**Figure 4:**
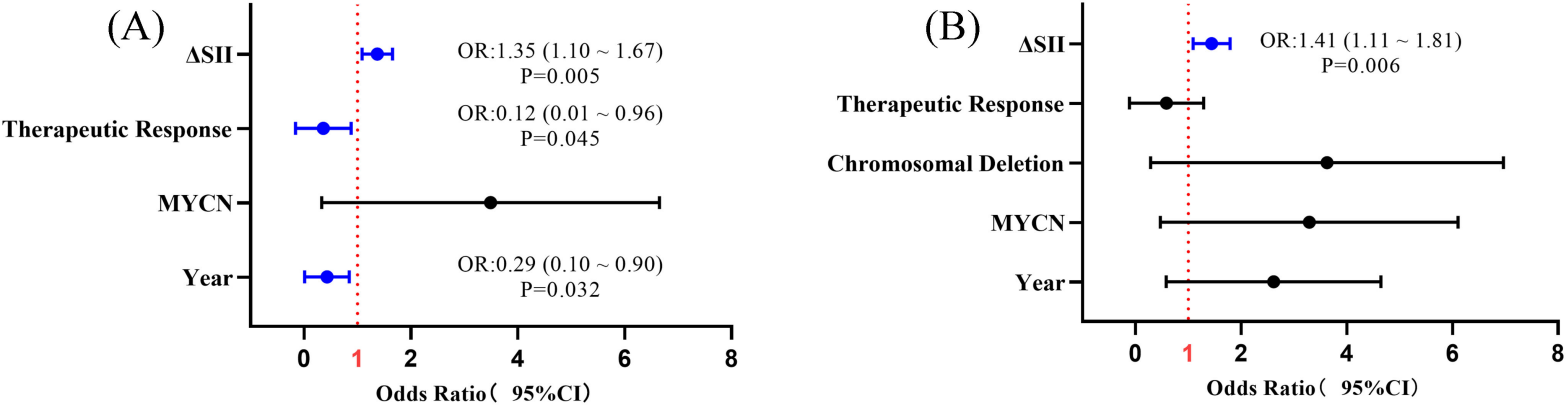
Forest plots of Cox proportional hazards model analysis for factors associated with overall survival (OS) and event-free survival (EFS). **(A)** Univariate and multivariate Cox regression analysis for OS. **(B)** Univariate and multivariate Cox regression analysis for EFS.

To explore the correlation between dynamic SII changes and survival prognosis in children with high-risk neuroblastoma, survival analysis and Cox proportional hazards model analysis were conducted. Survival curve analysis using the Kaplan-Meier method stratified patients based on the median ∆SII. Results (**Figure 3**) showed that the event-free survival (EFS) and overall survival (OS) of patients with lower ∆SII were significantly better than those with higher ∆SII (Log-rank test, P<0.001). Cox proportional hazards model analysis incorporating Year*, Sex, Pathologic, MYCN amplification, Chromosomal Deletion, Therapeutic Response, and ∆SII further revealed (**Tables 4**, **5**) that in univariate analysis, ∆SII, MYCN gene status, and treatment response were significantly associated with OS; ∆SII, MYCN gene status, Chromosomal Deletion, and treatment response were significantly associated with EFS (P<0.05). Multivariate analysis indicated that after adjusting for confounding factors, ∆SII had independent predictive value for OS (HR = 1.35, 95%CI: 1.10-1.67, P = 0.005) and EFS (HR = 1.41, 95%CI: 1.11-1.81, P = 0.006) (**Figure 4**). These results suggest that dynamic SII changes are an independent predictive factor for survival prognosis in children with high-risk neuroblastoma.

**Table 4:** Cox regression analysis of SII change rate for overall survival (OS).

Variables	Univariate analysis		Multivariate analysis	
	β (95%CI)	P	β (95%CI)	P
Year(month)*				
< 18	1.00 (Reference)		1.00 (Reference)	
≥ 18	0.85 (0.32 ~ 2.21)	0.733	0.29 (0.10 ~ 0.90)	0.032
Sex				
Male	1.00 (Reference)			
Female	0.94 (0.39 ~ 2.23)	0.880		
Pathologic				
Neuroblastoma	1.00 (Reference)			
Ganglioneuroblastoma, nodular	1.43 (0.52 ~ 3.95)	0.486		
MYCN				
No	1.00 (Reference)		1.00 (Reference)	
Yes	3.84 (1.62 ~ 9.10)	0.002	2.54 (0.92 ~ 7.02)	0.071
11q Deletion				
No	1.00 (Reference)			
Yes	2.44 (0.85 ~ 6.98)	0.097		
Therapeutic Response				
SD or PD	1.00 (Reference)		1.00 (Reference)	
CR or PR	0.09 (0.01 ~ 0.69)	0.020	0.12 (0.01 ~ 0.96)	0.045
ΔSII	1.47 (1.22 ~ 1.77)	<.001	1.35 (1.10 ~ 1.67)	0.005

CI, Confidence Interval; MYCN, MYCN gene amplification; 11q Deletion, Chromosome 11q deletion; SII, Systemic Immune-Inflammation Index; ΔSII, The change rate of the systemic inflammatory index (SII); CR, Complete Response; PR, Partial Response; SD, Stable Disease; PD, Progressive Disease. Year*, Age is presented as a category.

**Table 5:** Cox regression analysis of SII change rate for event-free survival (EFS).

Variables	Univariate analysis		Multivariate analysis	
	β (95%CI)	P	β (95%CI)	P
Year(month)*				
< 18	1.00 (Reference)			
≥ 18	1.01 (0.41 ~ 2.45)	0.988		
Sex				
Male	1.00 (Reference)		1.00 (Reference)	
Female	1.49 (0.67 ~ 3.35)	0.330	2.09 (0.90 ~ 4.86)	0.086
Pathologic				
Neuroblastoma	1.00 (Reference)			
Ganglioneuroblastoma, nodular	1.19 (0.44 ~ 3.25)	0.730		
MYCN				
No	1.00 (Reference)		1.00 (Reference)	
Yes	3.72 (1.63 ~ 8.49)	0.002	2.49 (0.96 ~ 6.42)	0.060
11q Deletion				
No	1.00 (Reference)		1.00 (Reference)	
Yes	2.88 (1.09 ~ 7.62)	0.033	2.60 (0.92 ~ 7.36)	0.072
Therapeutic Response				
SD or PD	1.00 (Reference)		1.00 (Reference)	
CR or PR	0.17 (0.04 ~ 0.74)	0.018	0.31 (0.07 ~ 1.39)	0.125
ΔSII	1.68 (1.30 ~ 2.17)	<.001	1.41 (1.11 ~ 1.81)	0.006

CI, Confidence Interval; MYCN, MYCN gene amplification; 11q Deletion, Chromosome 11q deletion; SII, Systemic Immune-Inflammation Index; ΔSII, The change rate of the systemic inflammatory index (SII); CR, Complete Response; PR, Partial Response; SD, Stable Disease; PD, Progressive Disease. Year*, Age is presented as a category.

**Figure 5:**
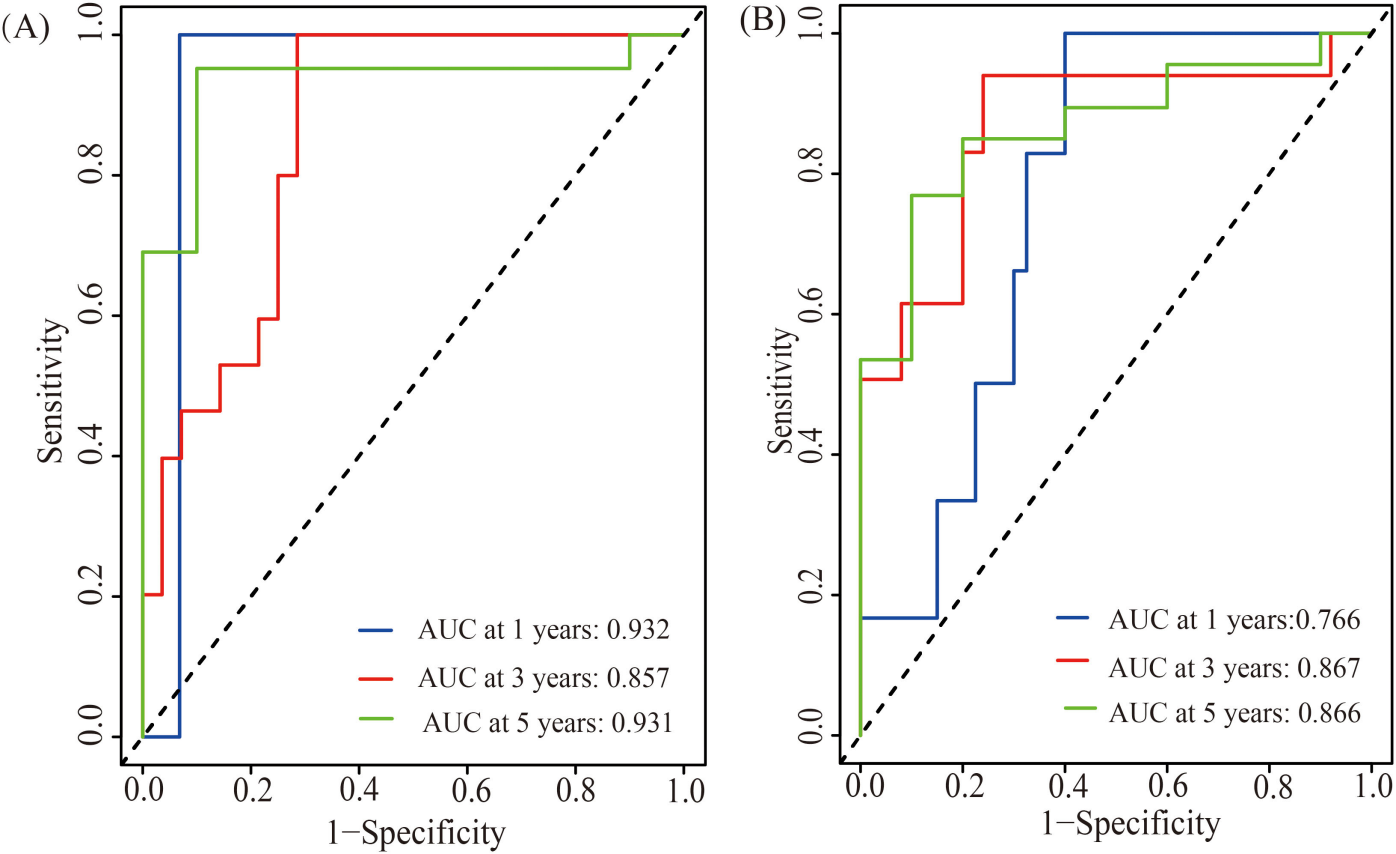
Time-dependent ROC curves for the predictive ability of the SII change rate for 1, 3, and 5-year OS **(A)** and EFS **(B)**.

Time-dependent ROC curve analysis was performed to evaluate the predictive ability of the SII change rate for survival outcomes in children with high-risk neuroblastoma. Results (**Figure 5**) indicated that the AUC values for the SII change rate in predicting 1-year, 3-year, and 5-year overall survival (OS) were 0.932, 0.857, and 0.931, respectively; for predicting 1-year, 3-year, and 5-year event-free survival (EFS), the AUC values were 0.766, 0.867, and 0.866, respectively. These results demonstrate that the SII change rate has high accuracy in predicting both short-term and long-term survival outcomes in children with high-risk neuroblastoma.

## Discussion

Neuroblastoma, a common pediatric solid tumor originating from neural crest cells, is highly heterogeneous with complex clinical presentations (15). High-risk neuroblastoma (HR-NB), with a poor prognosis and long-term survival rate of less than 50%, has always been a key focus and challenge in clinical research (16, 17). This study focused on the dynamic changes of the Systemic Immune-Inflammation Index (SII) during neoadjuvant chemotherapy (NAC), aiming to provide a new biomarker for treatment monitoring of HR-NB and optimize individualized treatment strategies.

In this study, significant differences were found in SII change patterns among different treatment response groups (P < 0.05). Patients in the CR/PR group showed a gradual decrease in SII during chemotherapy, while those in the SD/PD group exhibited no significant change or an upward trend. This suggests that dynamic SII changes may be closely related to tumor chemosensitivity. Spearman correlation analysis showed a significant positive correlation between the post-treatment SII change rate (∆SII) and the tumor diameter change rate (r=0.606, P<0.001). Further linear regression analysis indicated that ∆SII was an independent influencing factor of the tumor diameter change rate (β=0.07, 95% CI: 0.05-0.10, P < 0.001). Similarly, in logistic regression analysis with treatment response as the dependent variable, the OR value of ∆SII was 0.00 (95% CI: 0.00-0.03, P = 0.010), indicating that ∆SII is an independent predictive factor for chemosensitivity. These findings are consistent with previous literature reporting a close relationship between inflammatory marker changes and tumor treatment responses (18, 19). Demircan et al. found that in gastric cancer patients, SII changes before and after neoadjuvant chemotherapy were significantly associated with tumor regression grade (TRG) and survival period, and patients with ΔSII<0 were more likely to achieve good tumor responses and prognosis (20).

In the subsequent survival analysis, ∆SII was found to be an independent predictive factor for event-free survival (EFS) and overall survival (OS) in HR-NB patients (HRs were 1.35 and 1.41, respectively, P<0.05). Time-dependent ROC curve analysis also showed that ∆SII had high predictive accuracy for 1-year, 3-year, and 5-year survival outcomes (AUC values ranged from 0.766 to 0.932). This indicates that dynamically monitoring SII changes can provide important evidence for prognosis evaluation in HR-NB patients. Similar findings were reported in pancreatic cancer research, where changes in SII after neoadjuvant therapy were significantly associated with overall survival, and patients with decreased SII had better prognoses (21). The continuous activation of inflammatory responses may enhance tumor cell proliferation, survival, and invasive capabilities while inhibiting the immune system’s ability to clear tumor cells. Therefore, dynamic SII changes not only reflect the evolution of inflammatory status but may also directly impact tumor biology and treatment responses (22).

Systemic inflammatory responses play a complex and critical role in tumor progression. Inflammation, by modulating the tumor microenvironment (TME), promotes tumor angiogenesis, metastasis, and immune evasion, thereby influencing tumor growth and treatment responses (18, 19). Inflammatory cells and mediators in the TME coordinate a series of pro-inflammatory responses, which not only promote the proliferation and growth of malignant cells but also support immunomodulatory adaptive immune responses, reducing the efficacy of chemotherapeutic drugs (23, 24). For example, the infiltration of tumor-associated macrophages (TAMs) in localized and metastatic neuroblastoma is related to the expression levels of inflammatory genes and is negatively correlated with patient survival rates (25). Additionally, inflammatory responses can further promote tumor progression and drug resistance by affecting tumor cell metabolism and signaling pathways (23).

In recent years, systemic inflammatory markers based on peripheral blood cell counts have become powerful tools for prognosis evaluation due to their non-invasive and easily obtainable nature. SII, which integrates neutrophil, platelet, and lymphocyte counts, more comprehensively reflects the host’s inflammatory status and has demonstrated significant prognostic value in various adult and pediatric cancers (9–13, 26, 27). Neutrophils and platelets play pro-inflammatory roles in inflammatory responses, while lymphocytes primarily exert anti-inflammatory and immune surveillance functions. Therefore, an elevated SII often indicates enhanced inflammatory responses and suppressed immune function, which are closely related to tumor progression and poor prognosis.

The current International Neuroblastoma Risk Group (INRG) stratification system mainly relies on static indicators such as age, stage, and MYCN amplification, making it difficult to predict treatment responses in real time (28). This study, by dynamically monitoring SII changes, provides a simple, non-invasive, and real-time biomarker for the clinic. It helps to identify patients with poor treatment responses early on and adjust treatment plans promptly to improve treatment outcomes. The degree of tumor volume reduction during the early stages of NAC is closely related to prognosis, but imaging assessments have certain lag effects (5). In contrast, monitoring △SII can rapidly provide treatment response information in the early stages of chemotherapy, offering evidence for individualized treatment adjustments.

Although this study provides meaningful insights, several limitations must be considered. First, its retrospective, single-center design introduces potential selection bias and unmeasured confounders, such as undetected infections or concomitant medications. Second, the small sample size limits statistical power and generalizability, underscoring the need for large-scale, multi-center prospective validation.Third, the treatment strategy in this cohort did not include autologous stem cell transplantation (ASCT) or anti-GD2 immunotherapy, as these were not routinely available at our center during the study period. Although this differs from current international standard regimens for high-risk neuroblastoma, this limitation also provides a unique opportunity to evaluate the prognostic value of inflammatory markers in the context of chemotherapy alone—a reality in many resource-limited settings. Fourth, SII is a non-specific marker susceptible to non-malignant inflammation. Moreover, only pre- and post-NAC SII changes were analyzed. Future work should include frequent longitudinal measurements to better capture inflammatory dynamics. In future research, we will prospectively collect tumor tissue samples and employ multi-omics analyses to validate the associations between SII and specific tumor microenvironment (TME) characteristics.

In summary, this study is the first to explore the role of dynamic SII changes in NAC for HR-NB, revealing a significant association with treatment responses and prognosis. This not only deepens the understanding of the relationship between inflammation and tumors but also offers a new perspective for precision treatment and prognosis evaluation in HR-NB.

## Data Availability

The raw data supporting the conclusions of this article will be made available by the authors, without undue reservation.

## References

[1] Castleberry RP. Neuroblastoma. Eur J Cancer. (1997) 33:1430–7. doi: 10.1016/s0959-8049(97)00308-0, PMID: 9337686

[2] Maris JM Hogarty MD Bagatell R Cohn SL. Neuroblastoma. Lancet. (2007) 369:2106–20. doi: 10.1016/S0140-6736(07)60983-0, PMID: 17586306

[3] Cohn SL Pearson ADJ London WB Monclair T Ambros PF Brodeur GM. The International Neuroblastoma Risk Group (INRG) classification system: an INRG Task Force report. J Clin Oncol. (2009) 27:289–97. doi: 10.1200/JCO.2008.16.6785, PMID: 19047291 PMC2650388

[4] Davidoff AM Fernandez-Pineda I Santana VM Shochat SJ. The role of neoadjuvant chemotherapy in children with Malignant solid tumors. Semin Pediatr Surg. (2012) 21:88–99. doi: 10.1053/j.sempedsurg.2011.10.010, PMID: 22248974

[5] Yoo SY Kim J-S Sung KW Jeon TY Choi JY Moon SH. The degree of tumor volume reduction during the early phase of induction chemotherapy is an independent prognostic factor in patients with high-risk neuroblastoma. Cancer. (2013) 119:656–64. doi: 10.1002/cncr.27775, PMID: 22952047

[6] Kang H Lee HY Lee KS Kim J-H. Imaging-based tumor treatment response evaluation: review of conventional, new, and emerging concepts. Korean J Radiol. (2012) 13:371–90. doi: 10.3348/kjr.2012.13.4.371, PMID: 22778559 PMC3384819

[7] Diakos CI Charles KA McMillan DC Clarke SJ. Cancer-related inflammation and treatment effectiveness. Lancet Oncol. (2014) 15:e493–503. doi: 10.1016/S1470-2045(14)70263-3, PMID: 25281468

[8] Hu B Yang X-R Xu Y Sun Y-F Sun C Guo W. Systemic immune-inflammation index predicts prognosis of patients after curative resection for hepatocellular carcinoma. Clin Cancer Res. (2014) 20:6212–22. doi: 10.1158/1078-0432.CCR-14-0442, PMID: 25271081

[9] Kunc M Gabrych A Dulak D Hasko K Styczewska M Szmyd D. Systemic inflammatory markers and serum lactate dehydrogenase predict survival in patients with Wilms tumour. Arch Med Sci. (2022) 18:1253–61. doi: 10.5114/aoms/125543, PMID: 36160344 PMC9479718

[10] Chen J-H Zhai E-T Yuan Y-J Wu K-M Xu J-B Peng J-J. Systemic immune-inflammation index for predicting prognosis of colorectal cancer. World J Gastroentero. (2017) 23:6261–72. doi: 10.3748/wjg.v23.i34.6261, PMID: 28974892 PMC5603492

[11] Huang H Liu Q Zhu L Zhang Y Lu X Wu Y. Prognostic value of preoperative systemic immune-inflammation index in patients with cervical cancer. Sci Rep-uk. (2019) 9:3284. doi: 10.1038/s41598-019-39150-0, PMID: 30824727 PMC6397230

[12] Huang Y Gao Y Wu Y Lin H. Prognostic value of systemic immune-inflammation index in patients with urologic cancers: a meta-analysis. Cancer Cell Int. (2020) 20:499. doi: 10.1186/s12935-020-01590-4, PMID: 33061851 PMC7552553

[13] Terasaki F Sugiura T Okamura Y Ito T Yamamoto Y Ashida R. Systemic immune-inflammation index as a prognostic marker for distal cholangiocarcinoma. Surg Today. (2021) 51:1602–9. doi: 10.1007/s00595-021-02312-7, PMID: 34142236

[14] Krystal J Foster JH. Treatment of high-risk neuroblastoma. Children (Basel). (2023) 10:1302. doi: 10.3390/children10081302, PMID: 37628301 PMC10453838

[15] Kk M Jm M GS A N Cl M LD Wa W. Neuroblastoma. Nat Rev Dis Primers. (2016) 2:1–21. doi: 10.1038/nrdp.2016.78, PMID: 27830764

[16] Jm M Md H RB Sl C. Neuroblastoma. Lancet (London England). (2007) 369:2106–20. doi: 10.1016/S0140-6736(07)60983-0, PMID: 17586306

[17] Smith V Foster J. High-risk neuroblastoma treatment review. Children (Basel). (2018) 5:114. doi: 10.3390/children5090114, PMID: 30154341 PMC6162495

[18] Greten FR Grivennikov SI. Inflammation and cancer: triggers, mechanisms, and consequences. Immunity. (2019) 51:27–41. doi: 10.1016/j.immuni.2019.06.025, PMID: 31315034 PMC6831096

[19] Crusz SM Balkwill FR. Inflammation and cancer: advances and new agents. Nat Rev Clin Oncol. (2015) 12:584–96. doi: 10.1038/nrclinonc.2015.105, PMID: 26122183

[20] Demircan NC Atcı MM Demir M Işık S Akagündüz B. Dynamic changes in systemic immune-inflammation index predict pathological tumor response and overall survival in patients with gastric or gastroesophageal junction cancer receiving neoadjuvant chemotherapy. Asia Pac J Clin Oncol. (2023) 19:104–12. doi: 10.1111/ajco.13784, PMID: 35538045

[21] Tan Y Hu B Li Q Cao W. Prognostic value and clinicopathological significance of pre-and post-treatment systemic immune-inflammation index in colorectal cancer patients: a meta-analysis. World J Surg Oncol. (2025) 23:11. doi: 10.1186/s12957-025-03662-z, PMID: 39806457 PMC11731527

[22] Zheng C Liu S Feng J Zhao X. Prognostic value of inflammation biomarkers for survival of patients with neuroblastoma. CMAR. (2020) 12:2415–25. doi: 10.2147/CMAR.S245622, PMID: 32280277 PMC7132027

[23] Zhao H Wu L Yan G Chen Y Zhou M Wu Y. Inflammation and tumor progression: signaling pathways and targeted intervention. Sig Transduct Target Ther. (2021) 6:263. doi: 10.1038/s41392-021-00658-5, PMID: 34248142 PMC8273155

[24] Hibino S Kawazoe T Kasahara H Itoh S Ishimoto T Sakata-Yanagimoto M. Inflammation-induced tumorigenesis and metastasis. IJMS. (2021) 22:5421. doi: 10.3390/ijms22115421, PMID: 34063828 PMC8196678

[25] Hadjidaniel MD Muthugounder S Hung LT Sheard MA Shirinbak S Chan RY. Tumor-associated macrophages promote neuroblastoma via STAT3 phosphorylation and up-regulation of c-MYC. Oncotarget. (2017) 8:91516–29. doi: 10.18632/oncotarget.21066, PMID: 29207662 PMC5710942

[26] Zhang K Hua Y-Q Wang D Chen L-Y Wu C-J Chen Z. Systemic immune-inflammation index predicts prognosis of patients with advanced pancreatic cancer. J Transl Med. (2019) 17:30. doi: 10.1186/s12967-019-1782-x, PMID: 30658662 PMC6339361

[27] Yamashita S Iwahashi Y Miyai H Matsumura N Hagino K Kikkawa K. Usefulness of preoperative high systemic immune-inflammation index as a prognostic biomarker in patients who undergo radical cystectomy for bladder cancer: multicenter analysis. Diagnostics. (2021) 11:2194. doi: 10.3390/diagnostics11122194, PMID: 34943433 PMC8700357

[28] Tolbert VP Matthay KK. Neuroblastoma: clinical and biological approach to risk stratification and treatment. Cell Tissue Res. (2018) 372:195–209. doi: 10.1007/s00441-018-2821-2, PMID: 29572647 PMC5918153

